# Naringenin attenuates hepatitis B virus X protein-induced hepatic steatosis

**DOI:** 10.1186/s12906-017-2019-2

**Published:** 2017-11-28

**Authors:** Hung-Jen Lin, Ko-Li Ku, I-Hsin Lin, Chia-Chou Yeh

**Affiliations:** 1 School of Post-baccalaureate Chinese Medicine, Tzu Chi University, Hualien, Taiwan; 2 Department of Chinese Medicine, Dalin Tzu Chi Hospital, Buddhist Tzu Chi Medical Foundation, Chia-Yi, 62247 Taiwan; 3 School of Post-baccalaureate Chinese Medicine, China Medical University, Taichung, Taiwan; 4 Division of Chinese Internal Medicine, China Medical University Hospital, Taichung, Taiwan

**Keywords:** HBx, Naringenin, Hepatic steatosis, SREBP1c, LXRα, PPARγ, CD36, Adiponectin, aP2, FAS

## Abstract

**Background:**

Naringenin (Nar), a common dietary flavonoid abundantly present in fruits, vegetables, and Chinese herbs, is believed to possess strong anti-inflammatory properties and to modulate hepatic apolipoprotein and lipid synthesis. However, there are no reports describing Nar’s effects on the hepatitis B virus protein X (HBx) -induced hepatic steatosis, and the detailed molecular mechanisms of the compound’s effects are still unclear.

**Methods:**

Nar was administered by oral gavage to HBx-transgenic mice from 4 to 6 weeks of age. Mice were sacrificed after 14 days of once-daily naringenin administration. Liver tissues and sera were collected for histopathology and biochemical analysis.

**Results:**

Nar counteracted hepatic lipid accumulation and liver dysfunction in HBx-transgenic mice. In addition, Nar significantly decreased expression of adipogenic and lipogenic genes in mice, suggesting that the compound may have therapeutic effects in the early stages of HBx-mediated hepatic steatosis. These results indicated that naringenin inhibits HBx-induced expression of hepatic adipogenic and lipogenic genes through suppression of HBx-induced gene expression, including decreases in the transcriptional activity of SREBP1c, LXRα, and PPARγ in HBx-trangenic mice and HBx-transfected HepG2 cells.

**Conclusions:**

Results from this study suggested that Nar may serve as a therapeutic agent for preventing HBx-infected hepatic steatosis in humans.

## Background

The hepatitis B virus (HBV) is strongly associated with hepatitis, liver cirrhosis, and hepatocellular carcinoma (HCC) [1–4]. Fatty liver disease has been recognized as a risk factor for HCC development [5]. The prevalence of hepatic steatosis has been shown to be associated with chronic HBV infection [6]. The gene encoding the HBV X protein (HBx) is located at the 3′ end of the single-stranded portion of the HBV genome; this segment integrates into host DNA during hepatocellular regeneration after each out-break of hepatitis, resulting in increased intracellular accumulation of HBx [7]. Accumulation of HBx can cause lipid accumulation in hepatocytes [6] and in HBx-transgenic mice [8]. The induction of pathogenesis of hepatic steatosis by HBV is mediated by the sterol regulatory element binding protein 1 (SREBP-1) and the peroxisome proliferator-activated receptor (PPAR) [6], resulting in transcriptional activation via the PI3K/AKT/PTEN pathway or by protein–protein interactions. The liver X receptor (LXR) controls genes that encode proteins associated with de novo lipogenesis, including SREBP1c, ACC1 (acetyl-CoA carboxylase 1), and FAS (fatty acid synthase) [9]. In the liver, LXR also controls the transcription of the gene encoding cholesterol 7-α-hydroxylase 1 (CYP7A1), a protein that is involved in the conversion of cholesterol into bile acids [10].

Many herbals and phytochemicals have been considered as potential therapeutic agents for the treatment of HCC [11–16]. Naringenin (Nar) is a common dietary flavonoid that is plentiful in fruits and vegetables. The compound also is abundant in herbs [17]. Several studies have shown that Nar possesses a variety of pharmacological effects, such as antioxidant activity [18], anti-inflammatory activities [19], anti-tumour-invasion activity [20], and regulation of apolipoprotein and lipid synthesis [21]. Nar counteracts the induction of lipogenesis genes in rats maintained on a high-fat diet or in a fasted-like state [22, 23]. However, there are no reports (to our knowledge) on the mechanism of Nar’s effects on HBx-induced hepatic steatosis. Therefore, in the present study, we investigated the potential therapeutic effects of Nar on HBV-associated liver damage and fatty liver accumulation in HBx-transgenic mice.

## Methods

### Reagents

#### HBx-transgenic mice

The HBx-transgenic mouse model was obtained from Professor Ting-Fen Tsai, National Yang Ming University. The Tsai laboratory constructed this model by introducing (into the C57BL/6 background) a copy of the HBx-encoding gene under the control of the liver-specific albumin promoter [24]. In the present study, all of the animal experiments used male mice of the line A106 in the C57BL/6 background [25]. The animals were housed (5 per cage) in a temperature-controlled room with an uninterrupted 12-h/12-h light/dark cycle. Animals were provided with free access to tap water and food throughout the experiments. All mouse manipulations were performed according to a study-specific protocol (No. 1041001–1) approved by the Institutional Animal Care and Use Committees of the Dalin Tzu Chi Hospital (Chia-Yi, Taiwan).

### Nar administration

To study the therapeutic effect of Nar on the fatty liver and early stage of liver pathogenesis, four-week-old HBx-transgenic male mice were randomly assigned to three groups of five animals each. Naringenin (CAS Number 67604-48-2 from Sigma R5010; Nar) was of >98% purity, dissolved in 100% ethanol at a concentration of 50 mM and stored at 20 °C. 30 mg/kg/d of Nar was dissolved in H2O and delivered to the mice by oral administration using a feeding needle once a day. Nar administered to the mice over 14 days of once-daily oral gavage (p. o.) using a feeding needle, at a dose 30 mg/kg. Mice were sacrificed under general anesthesia by inhalation of 3% isoflurane at 14 days after Nar administration. At necropsy, liver tissues and sera were collected for histopathology and biochemical analysis. The sera were frozen at −80 °C pending assay; separate segments of each liver sample were fixed in formalin or flash frozen at −80 °C pending further processing.

### Histopathologic analysis

Liver specimens were processed using standard methods. The formalin-fixed liver samples were embedded in paraffin and sectioned; the resulting slides were stained with hematoxylin–eosin (H&E) and evaluated by light microscopy. Frozen liver samples were sectioned using a cryotstat; the resulting slides were stained with Oil red-O and evaluated by light microscopy to detect fat accumulation [8].

### Cell culture

Cells of the human hepatoma cell line HepG2 (Bio Resource Collection and Research Centre, Taiwan) were propagated in Dulbecco’s Modified Eagle Medium (DMEM; Life Technologies, Gaithersburg, MD, USA) supplemented with 10% fetal bovine serum (HyClone, Logan, UT, USA). The cells were transiently transfected with 5 μg of plasmid DNA using SuperFect Transfection Reagent (Qiagen, Valencia, CA, USA). The resulting stable HepG2 transfectants (HepG2-HBx), which carried a construct providing doxycycline (DOX) -inducible expression of a HBx-green fluorescent protein (GFP) fusion protein (HBx-GFP), were maintained in complete Minimal Essential Medium (MEM) supplemented with 100 μg/mL G418 and 50 μg/mL hygromycin. HepG2-HBx cells were resuspended in medium (100 μL/well) in 96-well plates and cultured with or without DOX and various concentrations of Nar. The viability of the treated HepG2-HBx cells was determined by MTT assay.

### RNA isolation and RT-PCR analysis

Total RNA was isolated from mouse tissues and stable HepG2-HBx cells using TRIzol Reagent (Life Technology). Gene expression was quantified using semi-quantitative reverse transcription-polymerase chain reaction (PCR) analysis as described previously [26]. The reverse transcription step was performed with 2 μg of total RNA using oligo-d(T) as primer and Superscript III reverse transcriptase (Invitrogen Life Technologies). Real-time reverse transcription PCR (RT-PCR) analyses were conducted on a Roche LightCycler 480 instrument using a TaqMan probe. All amplifications were performed in triplicate for each RNA sample, and all experiments were carried out using RNA samples from 3 separate mice; total input cDNA was equalized for all samples. RT-PCR results for each reaction were normalized against an internal control consisting of the transcription of a house-keeping gene (encoding hypoxanthine phosphoribosyltransferase (HPRT)). The PCR primers are listed in Table 1. The intensity of band on the gel was calculated by Gel-Pro® Analyzer (Media Cybernetics, Inc., Silver Spring, MD).

**Table 1:** Oligonucleotide primer sets and conditions used for semi-quantitative RT-PCR

Primer-name	Sequence
PPARyl-Forward	5′-TCTCCATGACAGACATGGACA-3’
PPARyl-Reverse	5′-GTCAGG CTGTTG GTCTCACA-3’
PPARy2-Forward	5′-GGGTGAAACTCTGGGAGATTCTC-3’
PPARy2-Reverse	5′-TCAGCAACCATTGGGTCAG-3’
PPARa-Forward	5′-GCAGCTCGTACAGGTCATCA-3’
PPARa-Reverse	5′-ACTGCCGTT GT CT GT CACT G-3’
SREBP-lc-Forward	5′-TTGTGGAGCTCAAAGACCTG-3’
SREBP-lc-Reverse	5′-TGCAAG AAGCGG AT GT AGTC-3’
LXRa-Forward	5′-TCCTACACGAGGATCAAGCG-3’
LXRa-Reverse	5′-AGTCGCAATGCAAAGACCTG-3’
GAPDH-Forward	5′-AGAACATCATCCCTG CAT CC-3’
GAPDH-Reverse	5′-CACATTGGGGGTAGGAACAC-3’
HBx-Forward	5′-TgTgCT gCCAACTggAT CCTg-3’
HBx-Reverse	5′-CCAATTTATgCCTACAgCCTCC-3’

### Preparation of nuclear extracts and electrophoretic mobility shift assay (EMSA)

HepG2-HBx cells were treated with 10–40 μM naringenin. Nuclear extracts were prepared as described previously [27]. Biotinylated electrophoretic mobility shift assays (EMSAs) were performed as previously described [28].

### Western blot analysis

Liver tissues from each mouse were homogenized at 4 °C in extraction buffer (100 mM Tris-HCl, pH 7.4, 5 mM EDTA, 50 mM NaCl, 50 mM sodium pyrophosphate, 50 mM NaF, 100 mM orthovanadate, 1% Triton X-100, 1 mM phenylmethanosulfonylfluoride, 2 mg/mL aprotinin, 1 mg/mL pepstatin A, and 1 mg/mL leupeptin) and centrifuged at 3000×g for 15 min at 4 °C. HepG2-HBx cells were lysed in 250 μL of sample buffer (62.5 mM Tris-HCl, pH 6.8, 2% SDS, 10% glycerol, 50 mM dithiothreitol, and 0.1% bromophenol blue). The amount of proteins in the tissue lysate and cell lytsate were assayed using the Bradford assay. Proteins (40 μg/sample for homogenized tissue, 10 μg/sample for cell lysates) were separated by 10% sodium dodecyl sulfate-polyacrylamide gel electrophoresis (SDS-PAGE), and protein bands were transferred electrophoretically to nitrocellulose membranes. Membranes were probed with polyclonal antibodies against SREBP1 (Santa Cruz Biotechnology, Santa Cruz, CA), GFP, AKT, AKT-p, PI3K, PI3K-p, C/EBPα, and β-actin (as a loading control) (Cell Signaling Technology, Beverly, MA, USA). Bound antibodies were detected using peroxidase-conjugated anti-rabbit antibodies followed by chemiluminescence assay (ECL System; Amersham, Buckinghamshire, UK) and autoradiographic exposure. The intensity of band on the bolts was calculated by Gel-Pro® Analyzer (Media Cybernetics, Inc., Silver Spring, MD).

### Statistical analysis

Two-tailed one-way analysis of variance (ANOVA) was used to identify significant differences between the means (*p <* 0.05). If the means differed significantly, a post-hoc Tukey-Kramer test was used to compare the groups. Data are shown as the mean ± standard error of the mean (SEM).

## Results

### Naringenin promotes recovery from fatty liver in HBx-transgenic mice

**Fig. 1:**
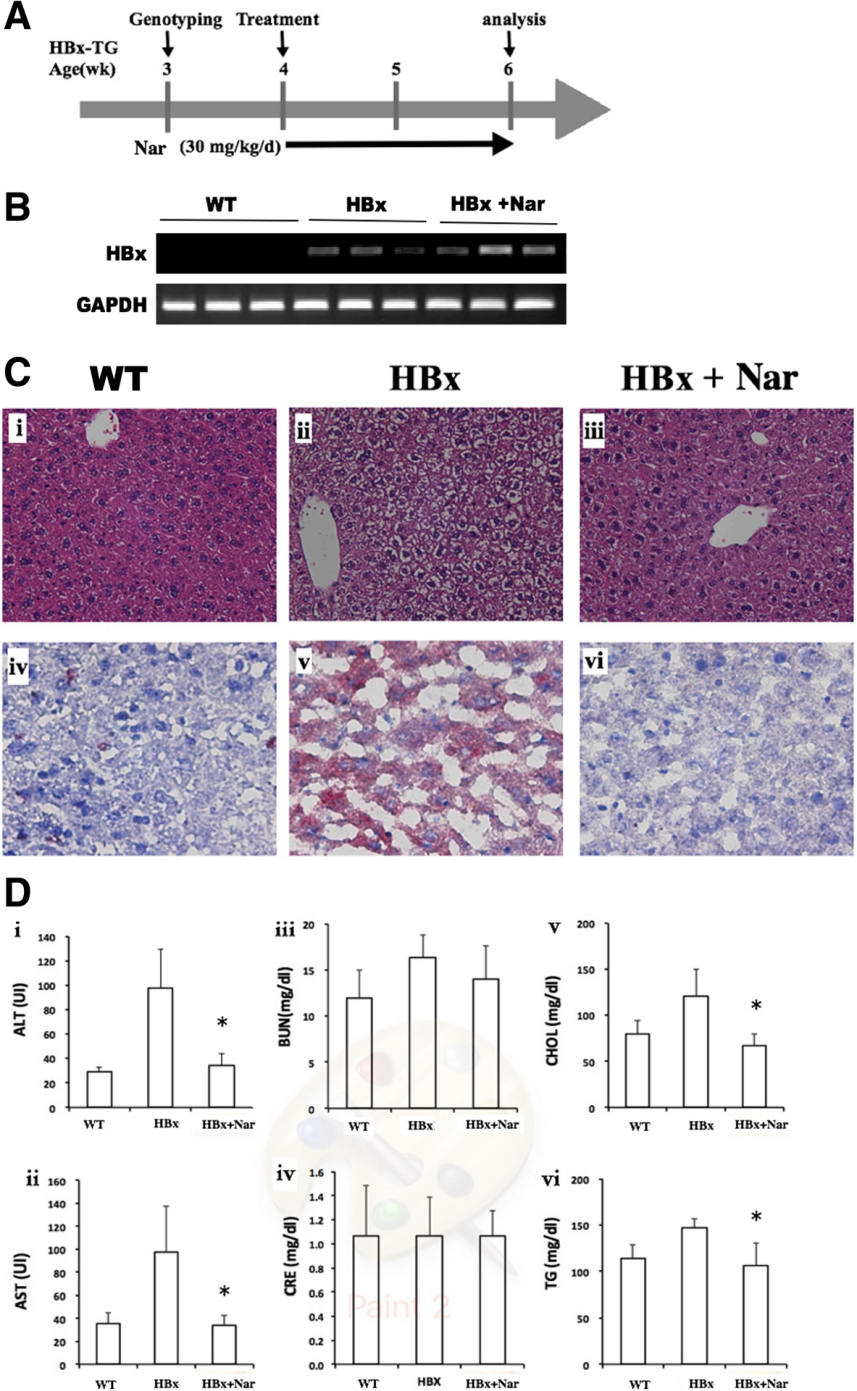
Treatment with naringenin (Nar) reverses fatty liver and early-stage liver damage in HBx-transgenic mice. **a** Schematic timeline of the treatment protocol. Animals were genotyped at 3 weeks of age. Starting at 4 weeks of age, HBX-transgenic mice (*n* = 5 per group) were dosed by 14 days of once-daily oral gavage with Nar (30 mg/kg) or an equivalent volume of vehicle (phosphate-buffered saline, PBS); wild-type (WT) littermates (*n* = 5) were dosed on the same schedule with vehicle. **b** Representative micrographs of hematoxylin and eosin (H&E) –stained sections of liver sections from a vehicle-dosed WT mouse (i), a vehicle-dosed HBx-transgenic mouse (ii), and a Nar-dosed HBx-transgenic mouse (iii), or representative micrographs of Oil red-O–stained sections of liver sections from animals of the same groups [(iv), (v), and (vi), respectively]. Original magnification, H&E 200×, oil red-O staining, 400×. **c** The levels of serum parameters for liver function [alanine aminotransferase (ALT) (i) and aspartate aminotransferase AST (ii)], renal function [blood urea nitrogen (BUN) (iii) and creatinine (Cre) (iv)], and lipid chemistry [total cholesterol (CHOL) (v) and triglycerides (TG) (vi)] were measured in vehicle-dosed WT mice, vehicle-dosed HBx-transgenic mice, and Nar-dosed HBx-transgenic mice. Values are expressed as mean ± SD. #, *p* < 0.05 between vehicle-treated WT and HBx-transgenic mice, *, *p* < 0.05 between HBx-transgenic vehicle- and Nar-treated mice

To investigate the effects of Nar (30 mg/kg/d, which corresponds to a daily dose that would be feasible in human) on HBx-induced hepatic lipid accumulation, we assessed the hepatic lipid content of liver tissues from HBx-transgenic mice. At an early stage of the HBx-mediated disease, 6-week-old vehicle-dosed HBx-transgenic mice exhibited liver pathology including fat accumulation, ballooning of the hepatocytes, and abnormal arrangements of the sinusoid [Fig. 1b(ii)]. Oral administration of Nar (30 mg/kg/d) to HBx-transgenic mice resulted in decreased liver damage and significant recovery of liver pathology [Fig. 1b (iii)] compared with the WT control [Fig. 1b (i)]. Oil-red O staining of liver sections showed that fatty liver was no longer seen in the HBx-transgenic mice administered Nar for 14 days [Fig. 1b (vi)]. There was no significant difference in the body weight or in the ratio of liver to body weight in the HBx-transgenic mice with or without Nar (data not shown).

The values for serum ALT and AST in the HBx-transgenic mice were significantly decreased after 14 days of Nar dosing (compared to control HBx mice) [Fig. 1c (i, ii)]. Notably, no significant difference was detected in the serum BUN and creatinine values in the HBx-transgenic animals with and without Nar treatment [Fig. 1c (iii, iv)]. Consistent with those results, the serum cholesterol and TG levels were significantly elevated in HBx-transgenic mice compared to WT mice and to HBx-transgenic mice that received Nar treatment [Fig. 1c (v, vi)]. Thus, our results showed that Nar treatment attenuated fatty liver pathology in the HBx-transgenic mice, indicating that this compound has therapeutic effects during the early stages of liver pathogenesis in this model.

### Effect of naringenin on HBx-induced expression of adipogenic and lipogenic genes in HBx-transgenic mice

**Fig. 2:**
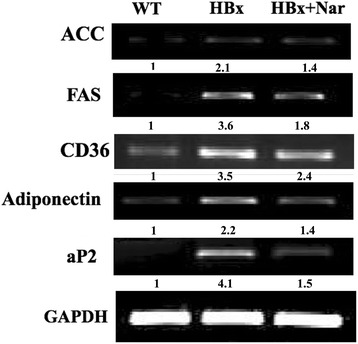
The effect of naringenin on the expression of adipogenic and lipogenic genes in 8-week-old HBx-transgenic mice. RT-PCR data are representative of 5 independent experiments

Next, we tested whether the overexpression of HBx is sufficient to decrease the hepatic lipid accumulation. The expression of adipogenic, and lipogenic–related genes were detected by RT-PCR in HBx-transgenic mice (Fig. 2). The levels of RNAs encoding FAS and ACC, downstream target genes of SREBP1 transcriptional control, were elevated in HBx-transgenic mice compared to the levels in WT mice. The levels of these transcripts in the HBx-transgenic mice were attenuated in HBx-transgenic animals dosed with Nar for 14 days. Similarly, transcripts encoding CD36, adiponectin, and aP2, all targets of PPARγ activity, accumulated to higher levels in HBx-transgenic mice, and this effect was attenuated in HBx-transgenic dosed with Nar for 14 days. However, levels of the mRNA encoding PPARα (and of PPARα protein) were not changed significantly in HBx-transgenic mice (compared to WT mice). Together, these observations indicated that Nar treatment counteracts HBx-induced expression of adipogenic and lipogenic genes, consistent with the decreased hepatic lipid accumulation observed with Nar dosing.

### Effect of naringenin on lipid accumulation and cell viability in HepG2-HBx cells

**Fig. 3:**
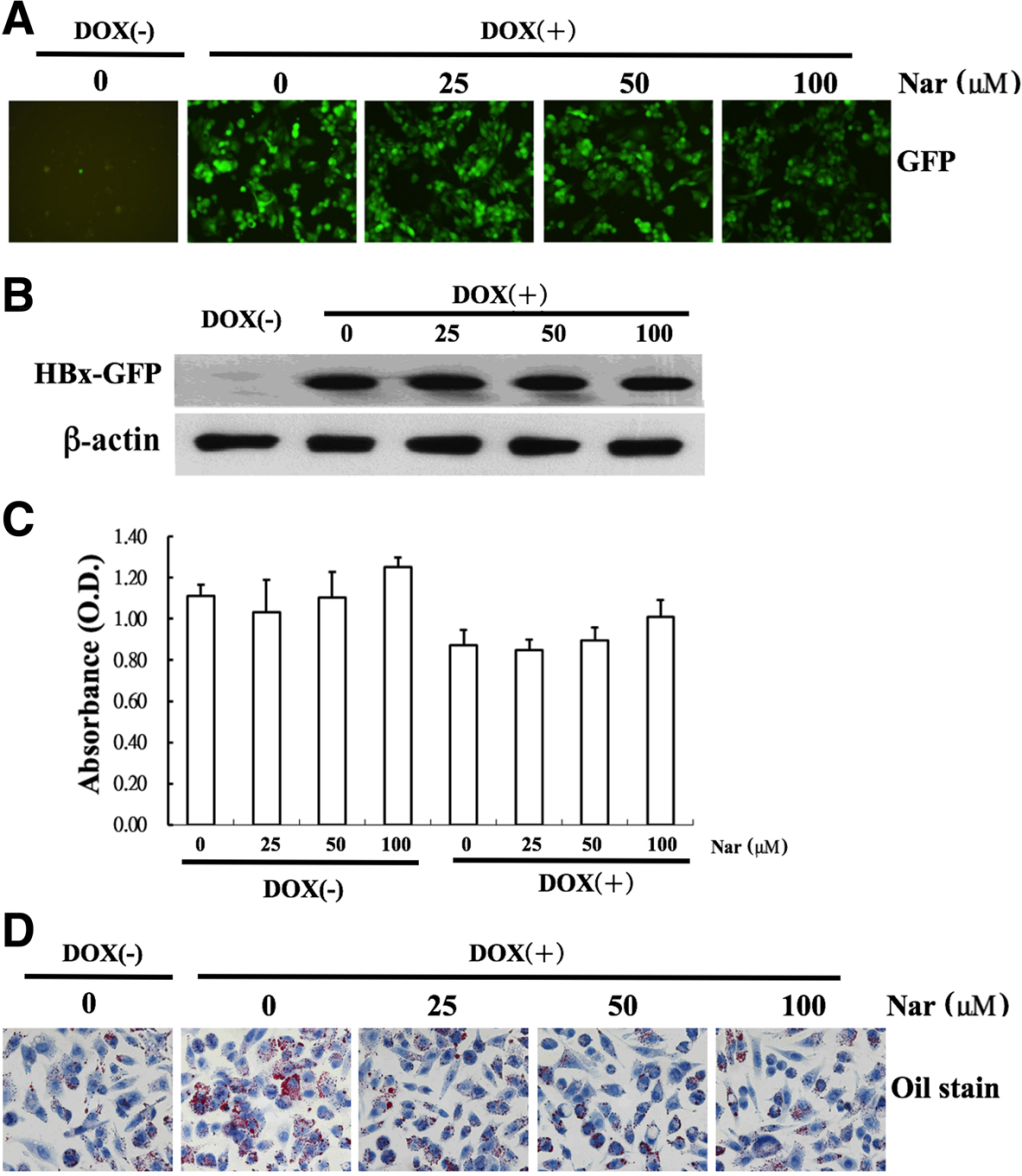
Establishment of constitutive HBx expression and Oil red-O staining in HepG2 cells. HepG2 cells were transfected with pRT-HBx-GFP. HepG2-HBx-GFP cells with and without DOX were observed under a fluorescence microscope (**a**). Expression of the HBx-GFP fusion protein in HepG2-HBx-GFP cells with and without DOX treatment (DOX(−) and DOX(+), respectively) was determined by western blot analysis using a GFP-specific antibody. β-actin was included as an internal control (**b**) After selection of G418-resistant colonies, cell proliferation with and without DOX treatment and with and without naringenin (Nar, at the indicated concentrations) was assessed by MTT assay (**c**). HepG2-HBx-GFP cells with and without DOX were treated with or without Nar at the indicated concentrations for 24 h. Intracellular lipid droplets in HepG2 cells were stained with Oil Red O and visualized by light microscopy (Olympus, Tokyo, Japan) (**d**)

To examine the effect of Nar on lipid accumulation under in vitro conditions, we first confirmed the effect of DOX induction on the expression of a HBx-GFP fusion in hepatic (HepG2) cells. GFP-fusion-protein expression was monitored using fluorescence microscopy, which detects fluorescence of the GFP protein (Fig. 3a), and by western blot analysis using an anti-GFP antibody (Fig. 3b). Both techniques showed that the GFP-fusion protein was detected only in the presence of DOX, demonstrating that the DOX-regulated HBx-GFP fusion had been successfully transfected into the recipient cells. The cytotoxicity of the HBx-GFP construct and that of Nar treatment were evaluated using the MTT assay. No cytotoxic effects were observed in HepG2-HBx cells exposed to 25–100 μM Nar in the absence of DOX; cell numbers were slightly decreased in the presence of DOX (Fig. 3c). Lipid accumulation (as assessed by Oil Red-O staining) was significantly decreased in DOX-induced HepG2-HBx cells exposed to 50 μg/mL Nar (and in uninduced (no-DOX) HepG2-HBx cells) when compared to that in DOX-induced HepG2-HBx cells grown in the absence of Nar (Fig. 3a). Thus, Nar also counteracted the HBx-associated lipid accumulation in an in vitro model, without apparent cytotoxicity.

### Nar inhibits the expression of lipogenic genes in HBx-transgenic mice and HepG2-HBx cells

**Fig. 4:**
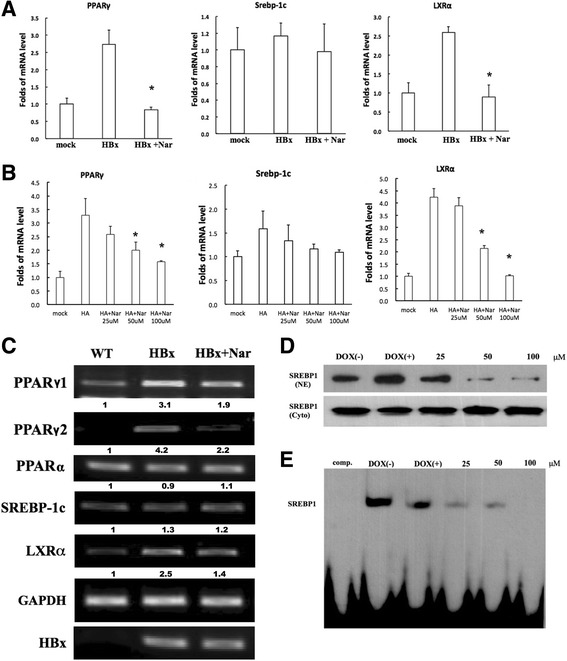
The effect of naringenin (Nar) on the expression of genes associated with lipogenesis in the HBx-transgenic livers. **a** Expression of the mRNAs encoding PPARγ, SREBP1-c, and LXRα in the livers of wild-type (WT) mice and in the livers of HBx-transgenic mice without and with 14 days of once-daily Nar treatment (30 mg/kg/d), as assessed by RT-PCR. **b** Expression of the mRNAs encoding PPARγ, SREBP1-c, and LXRα in HepG2 cells that had been mock-transfected (mock), or stably transfected with the HBx-GFP construct (HA) and grown in the presence of 0, 25, 50, or 100 μM Nar, as assessed by real-time PCR. **c** Repeat of the experiment portrayed in Panel A, this time using reverse transcription-PCR. Amplification products were electrophoresed, stained with ethidium bromide, and visualized using a transilluminator. **d** Nar attenuates HBx-induced nuclear translocation of the SREBP1 protein in HepG2-HBx cells. HepG2-HBx cells were grown in the absence of inducer (DOX(−)) or in the presence of inducer and Nar at 0 (DOX(+)), 25, 50, or 100 μM Nar. After 24 h of growth, cells were harvested, and the nuclear extract (NE) and cytoplasmic (Cyto) fractions were generated and processed for immunodetection of the SREBP1 protein as described in the Methods section. **e** Nar attenuates HBx-induced activation of the SREBP1 protein in HepG2-HBx cells. Cultures were grown as in Panel **d**, and samples collected after 24un hours were processed for biotinylated electrophoretic mobility shift assays as described in the Methods section. For Panels **a** and **b**, data are presented as mean ± SD from each group of 5 animals. Asterisk indicates a significant difference (*p* < 0.05 by two-tailed unpaired t test) comparing HBX mouse ± Nar or HepG2-HBx ± Nar. For Panels **c**, **d**, and **e**, reactions were performed with samples from individual mice or cultures; results are representative of those seen for biological replicates

**Fig. 5:**
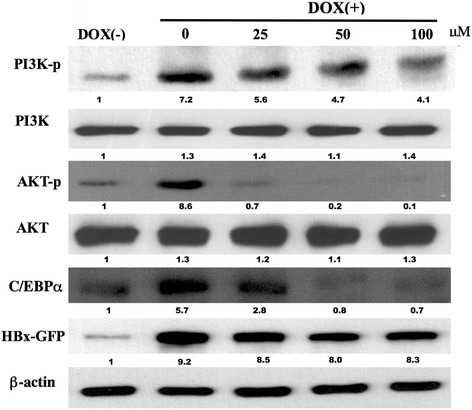
Effect of naringenin on HBx-induced AKT- signaling pathways. HepG2-HBx-GFP cells were pretreated with 0–100 μM naringenin before incubation with DOX for 45 min. Whole-cell lysates were then prepared and subjected to western blot analysis using antibodies specific for phosphorylated PI3K, AKT, and C/EBPα

To discover the molecular mechanisms underlying the beneficial effects of Nar on HBx-mediated fatty liver at the early stage of liver damage, we investigated the expression of lipogenic genes in liver by reverse transcriptase PCR and RT-PCR (Fig. 4a–c). Previous reports showed that the expression of HBx (in HBx-transgenic mice) induces expression of LXR, and of lipogenic proteins (e.g. Srebp1-c and PPARγ) encoded by genes regulated by LXR [9, 29] We observed that Nar dosing of HBx-transgenic mice decreased the expression of mRNAs encoding PPARγ and LXRα, but had no significant effect on *Srebp-1c* transcript levels (Fig. 4a). We confirmed these results in HepG2-HBx cells: exposure 50 or 100 μM Nar yielded significant decreases in the accumulation of *PPARγ* and *LXRα* mRNAs, while the effects on *Srebp-1c* transcript levels did not achieve significance (Fig. 4b). We further evaluated the effects of Nar by examining (separately) the nuclear translocation of Srebp1c and the DNA-binding activity of the protein. In control cells (grown without the DOX inducer), the majority of Srebp-1c was retained in cytoplasm; in contrast, the induction of HBx expression (by growth in the presence of Dox) resulted in increased translocation of Srebp-1c to the nucleus. Notably, growth in the presence of both DOX and Nar yielded nominally dose-dependent decreases in nuclear Srebp-1c (Fig. 4d). Similarly, biotinylated EMSAs showed that expression of HBx enhanced the DNA-binding activity of SREBP1, while Nar exposure yielded nominally dose-dependent decreases in this activity (Fig. 5b).

### Inhibitory effect of naringenin on HBx-induced activation of PI3K/AKT and C/EBPα

To further elucidate the mechanism of Nar activity, we examined the effect of the compound on crosstalk among the PI3K, AKT, and C/EBPα pathways in HepG2-HBx cells. Previous work showed that HBx can enhance *PPAR* gene expression through C/EBP**α** activation [6, 30]. Therefore, we examined whether the HBx-induced expression of C/EBP**α** was influenced by Nar. As expected, C/EBP**α** protein levels were increased in DOX-treated HepG2-HBx cells; additional exposure to Nar yielded apparently dose-dependent attenuation of C/EBP**α** protein accumulation (Fig. 4).

Previous work also showed that PPARγ promotes metabolic adaptations downstream of the PI3K/Akt pathway that favor hepatocyte steatosis [31]. Therefore, we investigated the effects of Nar on HBx-induced PI3K/Akt activity in HepG2-HBx cells. Western blot analysis revealed that the DOX-induced HBx expression resulted in apparent increases in the levels of phosphorylated PI3K and Akt (p-PI3K and p-Akt, respectively) compared to those in DOX-untreated controls, without obviously altering the accumulation of the proteins (phosphorylated + unphosphorylated) themselves. Exposure to Nar attenuated the amounts of the phosphorylated species in a nominally dose-dependent fashion (Fig. 5).

## Discussion

Hepatic steatosis (fatty liver) is associated with hepatitis virus infection, various drugs, and multiple genetic defects in energy metabolism. Fatty liver is an important factor in liver damage, and results in a further progression to cirrhosis and HCC [32]. HBx has been reported to be associated with HBV-related pathogenesis [33] and related to the development of hepatic steatosis in patients with HBV infections [4, 24]. As demonstrated in the present study, Nar exhibits therapeutic effects on the early stages of HBx-induced hepatic steatosis, attenuating fatty liver as assessed by histopathology, serum chemistry, and gene expression.

The accumulation of free fatty acids (FFAs) and cholesterol in the liver results in the production of reactive oxygen species (ROS) and tumour necrosis factor (TNFα) -mediated inflammation [34]. In the process of hepatic synthesis of endogenous fatty acids (FAs), ACC1 and FAS produce TGs, which are stored or rapidly metabolized. CD36 is the major FA transporter and enhances FFA uptake in hepatocytes [35]. LXRα, along with SREBP1c (the major isoform in the liver), affects the transcription of the genes encoding ACC1 and FAS, and that of its own gene, consequently stimulating hepatic lipogenesis [9, 36]. LXRα regulates the induction by HBx of SREBP1-mediated hepatic lipid synthesis and accumulation [9]. Separately, the transcriptional regulator PPARγ controls adipogenesis and plays a major role in the process of lipid storage. HBx also reinforces the transcriptional activity of PPARγ1 and γ2, resulting in increased expression of the *CD36* gene and adipogenic genes, including adiposin, aP2, and adiponectin [6]. HBx affects various lipid metabolic pathways and lipid deposition in liver. A previous study demonstrated that Nar significantly lowers serum triacylglycerol and cholesterol levels and decreases the expression of various lipogenic genes and SREBP-1c in HFD [23]. Separate work showed that Nar modulates the activity of PPARγ and LXRα, thereby downregulating the expression of FAS [22]. In the present study, we showed that Nar attenuates the HBx-induced accumulation of the transcripts encoding FAS, ACC1, CD36, adiponectin, aP2, PPAR1γ, PPARγ2, and LXRα in the in vivo model of HBx-transgenic mice. We further demonstrated, using the in vitro model of HepG2-HBx cells, that Nar exposure counteracts HBx-induced nuclear translocation and DNA-binding activity of SREBP.

Moreover, a previous study suggested that the activation of Akt is involved in the HBx-regulated process of survival and the activation of Srebp1c in the liver [37]. In hepatocytes, PPARγ may play a predominant role in the metabolic adaptation downstream of PI3K/Akt2 pathway, leading to steatosis [31] HBx also increases the expression of PPARγ, an effect thought to result from C/EBPα activation [6]. Our results demonstrated that Nar exposure results in a significant attenuation of the HBx-induced accumulation of p-Akt, p-PI3K, and C/EBPα in HepG2-HBx cells, suggesting molecular mechanisms whereby Nar counteracts lipid accumulation in hepatocytes both in vivo and in vitro. Specifically, Nar appears to suppress lipogenesis by decreasing LXRα-Srebp1c signaling and, thus, suppresses the expression of downstream genes (such as *ACC* and *FAS*) regulated by this pathway. This decrease in LXRa-Srebp1c signaling apparently attenuates hepatic PPARγ overexpression, thereby providing hepatic protection from FFA-mediated damage in HBx-transgenic mice dosed with Nar.

## Conclusions

**Fig. 6:**
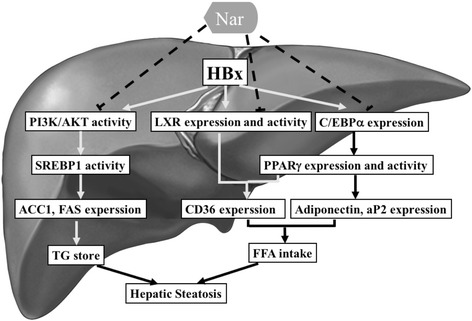
Schematic diagram illustrates the molecular mechanism of Nar attenuated HBx-induced liver steatosis

The present study demonstrated that Nar significantly reduced hepatic lipid accumulation and liver dysfunction in HBx-transgenic mice (Fig. 6). Furthermore, Nar significantly decreased expression of adipogenic and lipogenic genes in HBx-transgenic mice and in HepG2-HBx cell culture, suggesting that Nar may have chemotherapeutic effects on the early stages of HBx-induced hepatic steatosis. These results indicated that Nar may inhibit the HBx-induced expression of hepatic adipogenic and lipogenic genes by suppressing HBx-mediated gene expression and attenuating the transcriptional activity of LXRα, SREBP1c, and PPARγ in HBx-transgenic mice and stably transfected HepG2-HBx cells. Nar may serve as a therapeutic agent for preventing HBx-infected hepatic steatosis in humans.

## Abbreviations

ACC1: Acetyl-CoA carboxylase 1
EMSA: Electrophoretic mobility shift assay
FAS: Fatty acid synthase
HBV: Hepatitis B virus
HBx: Hepatitis B X protein gene
HCC: Hepatocellular carcinoma
LXRα: Liver X receptor alpha
Nor: Naringenin
PPAR: Peroxisome proliferator-activated receptor
SREBP1: Sterol regulatory element binding protein 1
